# Effects of Application of Pig Manure on the Accumulation of Heavy Metals in Rice

**DOI:** 10.3390/plants11020207

**Published:** 2022-01-14

**Authors:** Wenchong Lan, Chunxia Yao, Fan Luo, Zhi Jin, Siwen Lu, Jun Li, Xindong Wang, Xuefeng Hu

**Affiliations:** 1 School of Environmental and Chemical Engineering, Shanghai University, Shanghai 200444, China; lanwenchong@shu.edu.cn (W.L.); fanluo@shu.edu.cn (F.L.); jinzhi@shu.edu.cn (Z.J.); lsw1105@163.com (S.L.); lijun17@shanghai-electric.com (J.L.); 19722709wxd@shu.edu.cn (X.W.); 2 Laboratory of Quality and Safety Risk Assessment for Agro-Products (Shanghai), China Ministry of Agriculture, Institute for Agro-Food Standards and Testing Technology, Shanghai Academy of Agricultural Sciences, Shanghai 201403, China

**Keywords:** pig manure (PM), fungal culturing residues (FCR), rice, heavy metal, soils

## Abstract

Pig manure (PM) is often highly enriched in heavy metals, such as Cu and Zn, due to the wide use of feed additives. To study the potential risks of heavy metal accumulation in the soil and rice grains by the application of PM and other organic manure, a four-year field experiment was conducted in the suburb of Shanghai, southeast China. The contents of Cu, Zn, Pb, and Cd in the soils and rice plants by the treatments of PM and fungal culturing residues (FCR) show a trend of annual increase. Those in the soils and rice by the PM treatment are raised even more significantly. Cu and Zn contents in the soil and rice roots by the PM are significantly higher than those by the non-fertilizer control (CK) during the four years, and Pb and Cd also significantly higher than CK in the latter two years. Heavy metals taken up by the rice plants are mostly retained in the roots. Cu and Zn contents in the rice plants are in the decreasing order of roots > grains > stems > leaves, and Pb and Cd in the order of roots > stems > leaves > grains. Cu, Zn, Pb, and Cd contents in the soils by the PM treatment increase by 73%, 32%, 106%, and 127% on annual average, and those in the brown rice by 104%, 98%, 275%, and 199%, respectively. The contents of Cu, Zn, Pb, and Cd in the brown rice of the treatments are significantly correlated with those in the soils and rice roots (*p* < 0.05), suggesting the heavy metals accumulated in the rice grains come from the application of PM and FCR. Though the contents of heavy metals in the brown rice during the four experimental years are still within the safe levels, the risks of their accumulative increments, especially by long-term application of PM, can never be neglected.

## 1. Introduction

Excessive application of chemical fertilizers often leads to agricultural diffuse pollution, causing the eutrophication of surface water and contamination of groundwater [1]. In recent decades, the replacement of chemical fertilizers by organic manure has been highly encouraged in China. The number of live pigs attained 680 million in China in 2016 [2]. PM is not only an important source of organic manure but also a link to the circulation of the livestock-soil-plant system [3]. Long-term application of PM significantly increases the contents of organic matter and nutrients in soils [4].

To prevent pigs from infecting intestinal pathogen and supplement nutrients, some trace elements such as Cu, Zn, Fe, and Mn are often added into livestock feed [5], thus causing the enrichment of heavy metals in the PM [6,7]. According to the investigations, the rates of livestock manures with Cd and Pb content exceeding the safe limits of organic fertilizers are 12.3% and 2.6%, and those with Cu and Zn exceeding the limits are 53.9% and 45.7%, respectively [8]. Long-term application of livestock manures hence often leads to the accumulation of heavy metals in agricultural soils [9,10]. PM is particularly highly enriched in Cu and Zn [11,12,13,14,15,16,17]. These in the PM released from an intensively raising farm in China attain as high as 1726 mg/kg and 1506 mg/kg, respectively [18]. The application of PM often causes the contamination of heavy metals and antibiotics in soils [19,20].

Rice is the staple food of nearly half of the world’s population [21]. The rice grain, however, is inclined to accumulate Cd and other toxic metals, even growing on Cd-low soils. Therefore, it never seldom occurs that Cd content in the rice in the market exceeds the safe limit (0.2 mg/kg) [22,23]. Whether long-term application of PM influences the contents of Cd and other toxic heavy metals in rice grains should be paid close attention to.

In this study, a field experiment of application of PM and two FCR are carried out in the suburb of Shanghai for four consecutive years from 2017 to 2020. The contents of Cu, Zn, Pb, and Cd in the soils, rice plants, and grains by the different fertilizer treatments are detected to study the effects of the application of PM and FCR on the accumulation of toxic heavy metals in rice grains and to evaluate its potential edible risks.

## 2. Results

### 2.1. Effects of Application of PM and FCR on Soil Nutrients

#### 2.1.1. Organic Matter (OM) Content

**Figure 1:**
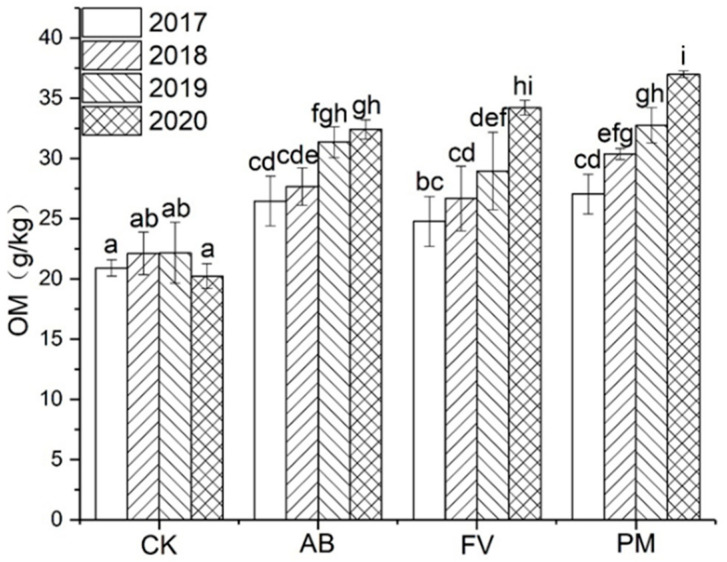
Effects of the different fertilizer treatments on the content of OM in the soils. All the data are expressed as means ± SD; *n* = 3. Different letters on data in the same group indicate a significant difference between them at *p* < 0.05 level.

The content of OM in the soils by the agaricusbisporus (AB), flammulinavelutipes (FV), and PM treatments shows a trend of annual increase (Figure 1). That in the AB, FV, and PM soils is significantly higher than CK (*p <* 0.05) in each experimental year, which increases by 38.0%, 34.2%, and 48.9% on annual average, respectively. Especially, OM content in the PM soil is significantly higher than that in AB, FV, and CK.

#### 2.1.2. Total N (TN) and Alkaline Hydrolyzed N (AN) Contents

**Figure 2:**
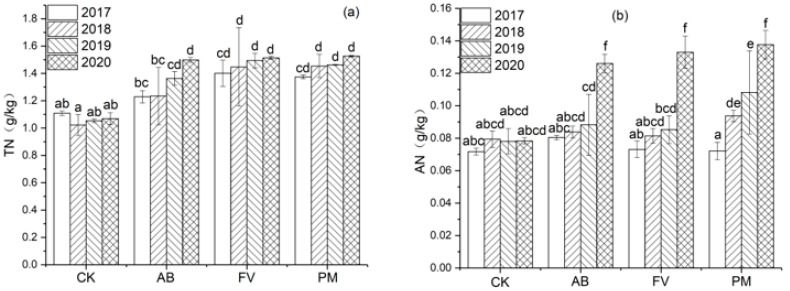
(**a**) Effects of the different fertilizer treatments on the content of TN in the soils; (**b**) Effects of the different fertilizer treatments on the content of AN in the soils. All the data are expressed as means ± SD; *n* = 3. Different letters on data in the same group indicate a significant difference between them at *p* < 0.05 level.

The contents of TN and AN in the AB, FV, and PM soils also increase annually (Figure 2). Compared with CK, these in the AB soil increase by 25.2% and 23.0% on annual average during the four experimental years; these in FV increase by 37.7% and 21.2%; these in PM increase by 36.8% and 33.8%, respectively. The contents of TN and AN in the PM soil in the fourth year are 1.1 and 1.9 times those in the first year and are higher than those in the AB and FV soils.

#### 2.1.3. Total P (TP) and Available P (AP) Contents

**Figure 3:**
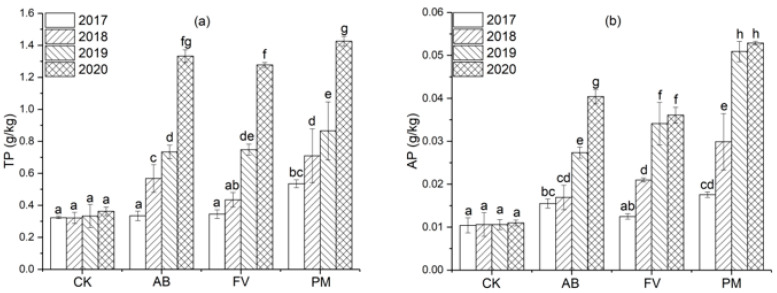
(**a**) Effects of the different fertilizer treatments on the content of TP in the soils; (**b**) Effects of the different fertilizer treatments on the content of AP in the soils. Data are expressed as means ± SD; *n* = 3. Different letters on data in the same group indicate a significant difference between them at *p* < 0.05 level.

The contents of TP and AP in the PM, AB, and FV soils also increase annually (Figure 3). Compared with CK, these in the AB soil increase by 121.6% and 135.0% on annual average during the four experimental years; these in the FV soil increase by 109.0% and 143.2%; these in the PM soil increase by 163.9% and 254.7%, respectively. TP and AP contents in the PM soil on the fourth year are 2.7 and 3.0 times those on the first year, respectively. The content of AP in the PM soil is significantly higher than that in the AB and FV soils (*p <* 0.05), and TP in the PM soil is also higher than in the AB and FV soils (*p* > 0.05) in the fourth year, suggesting that the PM application is more beneficial to P increase in the soil.

#### 2.1.4. Total K (TK) and Available K (AK) Contents

**Figure 4:**
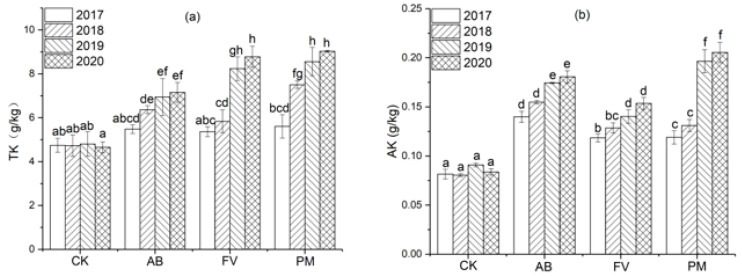
(**a**) Effects of the different fertilizer treatments on the content of TK in the soils; (**b**) Effects of the different fertilizer treatments on the content of AK in the soils. Data are expressed as means ± SD; *n* = 3. Different letters on data in the same group indicate a significant difference between them at *p* < 0.05 level.

The contents of TK and AK in the PM, AB, and FV soils also increase annually (Figure 4). During the four experimental years, the contents of TK and AK in the PM, AB, and FV soils are significantly higher than CK (*p <* 0.05). Compared with CK, moreover, these in the AB soil increase by 37.1% and 93.0% on annual average; these in the FV soil increase by 49.2% and 60.6%; these in the PM soil increase by 62.3% and 93.6%, respectively. TK and AK contents in the PM soil in the fourth year are 1.6 and 1.7 times those in the first year, respectively. The content of AK in the PM soil in the third and fourth years is significantly higher than that in the AB and FV soils (*p* < 0.05).

### 2.2. Effects of Application of PM and FCR on Rice Yield

**Figure 5:**
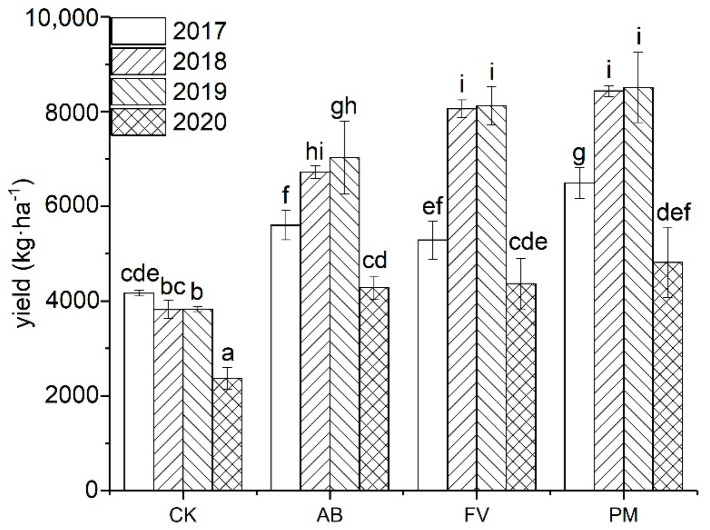
Yield of rice grains by the different fertilizer treatments during the four experimental years. Data are expressed as means ± SD; *n* = 3. Different letters on data in the same group indicate a significant difference between them at *p* < 0.05 level.

During the four experimental years, the yield of rice grain by the AB, FV, and PM treatments increases by 40.0%, 45.1%, and 49.8% on annual average, respectively (Figure 5). The effect of yield increase by the PM treatment is significantly higher than that of AB and FV. The grain yield of the three fertilizer treatments in the second and third years is significantly higher than that of the first year (*p* < 0.05). That of the fourth year, however, is significantly reduced, possibly related to unsuitable climate and severer incidence of rice pests and diseases.

### 2.3. Effects of Application of PM and FCR on the Accumulation of Heavy Metals in the Soil Plant System

#### 2.3.1. Heavy Metal Contents in the Soils

**Figure 6:**
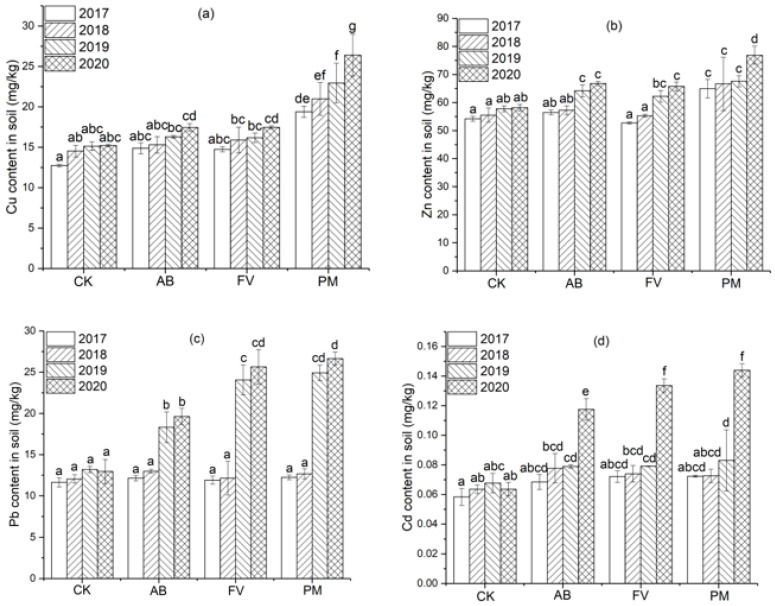
(**a**) Cu content in the soils by the different fertilizer treatments during the four experimental years; (**b**) Zn content in the soils by the different fertilizer treatments during the four experimental years; (**c**) Pb content in the soils by the different fertilizer treatments during the four experimental years; (**d**) Cd content in the soils by the different fertilizer treatments during the four experimental years. Data are expressed as means ± SD; *n* = 3. Different letters on data in the same group indicate a significant difference between them at *p* < 0.05 level.

During the four experimental years, the contents of Cu, Zn, Pb, and Cd in the PM, AB, and FV soils also show a trend of annual increase, which are mostly higher than CK (Figure 6). Especially, the contents of Cu and Zn in the PM soil during the four years are significantly higher than CK (*p* < 0.05) (Figure 6a,b), and Pb and Cd in the latter two years are also significantly higher than CK (*p* < 0.05) (Figure 6c,d).

During the four years from 2017 to 2020, Cu content in the PM soils is 1.5, 1.4, 1.5, and 1.7 times higher than CK (Figure 6a), and Zn content is 1.2, 1.2, 1.2, and 1.3 times higher than CK (Figure 6b), respectively. Pb content in the PM soil in the latter two years is 1.9 and 2.1 times higher than CK (Figure 6c), and Cd content is 1.2 and 2.3 times higher than CK (Figure 6d), respectively.

#### 2.3.2. Heavy Metal Contents in the Rice Roots

**Figure 7:**
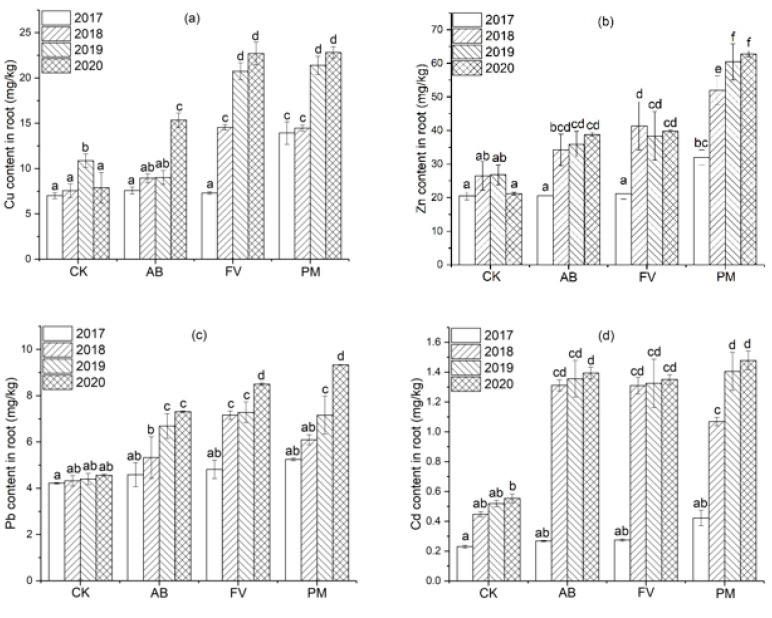
(**a**) Cu content in the rice roots by the different fertilizer treatments during the four experimental years; (**b**) Zn content in the rice roots by the different fertilizer treatments during the four experimental years; (**c**) Pb content in the rice roots by the different fertilizer treatments during the four experimental years; (**d**) Cd content in the rice roots by the different fertilizer treatments during the four experimental years. Data are expressed as means ± SD; *n* = 3. Different letters on data in the same group indicate a significant difference between them at *p* < 0.05 level.

The contents of Cu, Zn, Pb, and Cd in the rice roots of the PM, AB, and FV treatments all show an annual increasing trend and attain maximum values in the fourth year (Figure 7).

Cu content in the rice roots of the PM, AB, and FV treatments in the fourth year is mostly significantly higher than that in the earlier three years (*p <* 0.05) (Figure 7a). Especially, that of PM on the fourth year attains 22.82 mg/kg, which is 2.9, 1.5, and 1.1 times higher than that of CK, AB, and FV in the same year, respectively. During the four experimental years, the Cu content of the PM roots is significantly higher than that of CK and AB (*p <* 0.05). Meanwhile, Zn content in the rice roots of the three treatments in the latter three years is significantly higher than that in the first year (*p <* 0.05) (Figure 7b). That in the PM roots in the latter two years is significantly higher than in the first two years (*p* < 0.05). Zn content in the PM roots is significantly higher than that in CK, AB, and FV (*p <* 0.05) during the four years. That on the fourth year attains as high as 62.84 mg/kg, which is 3.0, 1.6, and 1.6 times higher than in CK, AB, and FV, respectively. Pb content in the PM roots in the latter two years is significantly higher than that in CK and AB (*p <* 0.05) (Figure 7c). That on the fourth year attains 9.33 mg/kg, which is 2.0, 1.3, and 1.1 times higher than that of CK, AB, and FV, respectively. Cd content in the PM roots during the four experimental years is higher than that of CK, AB, and FV (Figure 7d). That on the fourth year attains 1.48 mg/kg, which is 2.7 times higher than CK and 3.5 times higher than in the first year.

#### 2.3.3. Heavy Metal Content in the Rice Grains

**Figure 8:**
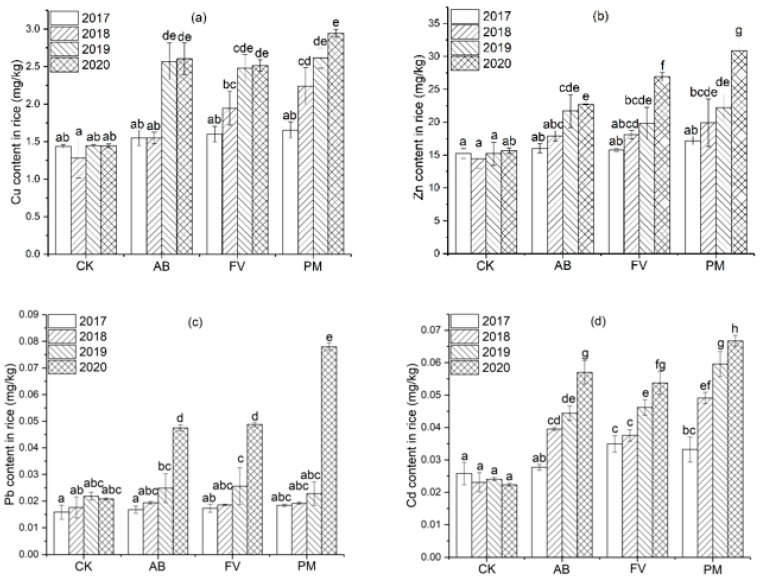
(**a**) Cu content in the brown rice by the different fertilizer treatments during the four experimental years; (**b**) Zn content in the brown rice by the different fertilizer treatments during the four experimental years; (**c**) Pb content in the brown rice by the different fertilizer treatments during the four experimental years; (**d**) Cd content in the brown rice by the different fertilizer treatments during the four experimental years. Data are expressed as means ± SD; *n* = 3. Different letters on data in the same group indicate a significant difference between them at *p* < 0.05 level.

The contents of Cu, Zn, Pb, and Cd in the brown rice of the three treatments also show a trend of annual increase, correlated with the annual increase in heavy metals in the soils and rice roots. Especially, these of PM during the four years are mostly significantly higher than CK (*p <* 0.05), and also mostly higher than these of AB and FV (Figure 8).

Cu content in the brown rice of PM is enhanced even more significantly and increases by 14.9%, 74.4%, 80.7%, and 103.6%annuallyduring the four years, compared with CK; that of AB increases by 7.5%, 20.6%, 77.3%, and 80.0%; that of FV increases by 11.3%, 51.4%, 71.2%, and 73.7%, respectively (Figure 8a).Zn content in the brown rice of PM increases by 12.9%, 37.8%, 46.1%, and 97.5%; that of the AB by 5.2%, 24.1%, 42.8%, and 45.3%; that of the FV by 3.6%, 25.5%, 30.2%, and 71.8%, respectively (Figure 8b).Pb content in the brown rice of the PM increases by 16.0%, 9.2%, 4.6%, and 274.9%; that of AB increases by 6.0%, 10.2%,14.0% and 128.3%; that of FV increases by 9.2%, 5.4%, 17.3%, and 134.3%, respectively (Figure 8c). Cd content of the brown rice of PM increases by 28.5%, 112.8%, 147.8%, and 198.9%; that of AB increases by 7.3%, 71.2%, 84.7%, and 155.4%; that of FV increases by 35.2%, 62.8%, 92.2%, and 140.7%, respectively (Figure 8d).

#### 2.3.4. Distribution of Heavy Metals in the Rice Plants

**Figure 9:**
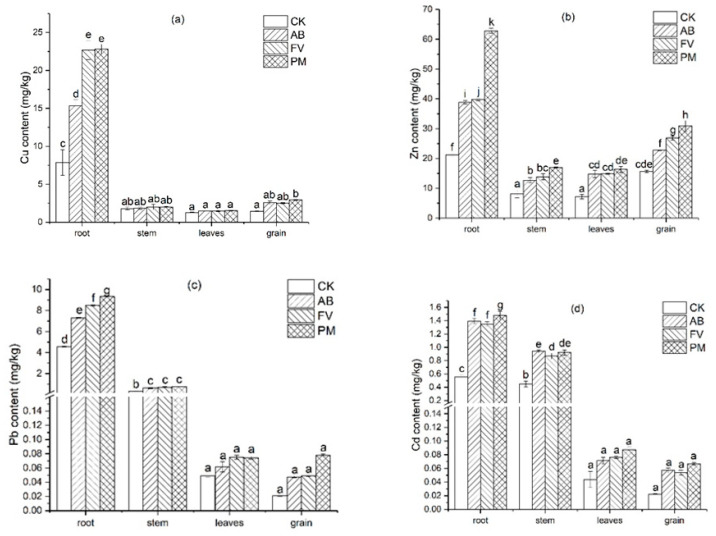
(**a**) Distribution of Cu content in the rice plants on the fourth experimental year; (**b**) distribution of Zn content in the rice plants on the fourth experimental year; (**c**) distribution of Pb content in the rice plants on the fourth experimental year; (**d**) distribution of Cd content in the rice plants on the fourth experimental year. Data are expressed as means ± SD; *n* = 3. Different letters on data in the same group indicate a significant difference between them at *p* < 0.05 level.

The distribution of Cu, Zn, Pb, and Cd in the different tissues of rice plants by the three treatments on the fourth year shows a similar rule (Figure 9): Cu content in the rice roots is significantly higher than that in the rice stems, leaves and brown rice (*p <* 0.05) (Figure 9a). Especially, Cu in the rice roots of PM is 11.5, 15.1, and 7.7 times that in the rice stems, leaves, and brown rice, respectively. Moreover, Cu content in brown rice is higher than that in rice stems and leaves (*p* < 0.05). Zn content in the rice roots and brown rice is also higher than that in the stems and leaves (Figure 9b). Especially, that of PM attains 62.84 and 30.90 mg/kg, respectively, which is significantly higher than in the stems and leaves (*p <* 0.05).

Pb content in the rice roots is significantly higher than that in the stems, leaves, and brown rice (*p <* 0.05), while that in the stems is also significantly higher than in the leaves and rice (*p <* 0.05) (Figure 9c). Cd content in the different tissue of rice plants shares a similar distribution with Pb (Figure 9d).

## 3. Discussion

### 3.1. Enhancements of Soil Nutrients by Application of PM and FCR

Soil fertility is gradually improved after the application of PM and FCR. The content of OM in the soils of AB, FV and PM increases by 38.0%, 34.2%, and 48.9% on annual average during the four years; TN, 25.1%, 38.4%, and 37.1%; AN, 23.0%, 21.2%, and 33.8%; TP, 121.6%, 109.4%, and 163.9%; AP, 135.0%, 143.2%, and 254.7%; TK, 37.1%, 49.2%, and 62.3%; AK 93.0%, 60.6%, and 93.6%, respectively. During the four experimental years, the content of OM in the soils of PM is significantly higher than that of AB, FV, and CK (Figure 1). The application of PM and FCR increases the content of soil OM and improves soil quality significantly [24,25]. The effects of PM application on the improvement of soil fertility are even more significant [26]. The excessive application of PM, however, often causes the accumulation of P in soils (Figure 3) and increases the risk of agricultural diffuse pollution [27].

Correlating with the annual enhancement of soil nutrients, the yield of rice grain of AB, FV, and PM increases by 40.0%, 45.1%, and 49.8%, on annual average, respectively. There is a significant positive correlation between the content of OM in the soils and the yield of rice grains (r = 0.886, *n* = 12; *p <* 0.01), further suggesting that the application of organic manure has contributed to the yield increase. The effects of yield increase by PM are even more significant as PM contains high content of nutrients [28,29]. Moreover, higher content of Zn in PM may also contribute to yield increase [30,31]. Over the first three years, the grain yield by the AB, FV, and PM continuously increases, and that of PM is significantly higher than of AB and FV, which is consistent with the previous results [32,33,34]. In the fourth year, the yield of rice grain of the treatments, however, is significantly reduced (Figure 5), which may be related to the unsuitable climatic conditions and high incidences of rice pests and diseases this year.

### 3.2. Accumulation of Heavy Metals in the Soils Caused by Application of PM and FCR

Feed additives commonly used in pig breeding often contain a certain amount of heavy metals, such as Cu, Zn, Fe, and Mn, which are used to prevent diseases, supplement nutrients and improve the growth of pigs [35,36,37]. The heavy metals added into feed are finally mostly released with dung [37,38]. PM is thus often highly enriched in Cu and Zn [39,40] and sometimes even contains toxic metals or metalloids such as Cd and As [41,42]. Toxic heavy metals in organic fertilizers that are made from animal manures therefore often exceed the safe limits [43]. The contents of Cu and Zn in 117 samples of organic fertilizers in northern China are 75.2 mg/kg and 581.9 mg/kg on average, respectively [44]. Cu content in the PM used in this study is 5.5 and 20.8 times that of FV and AB, and Zn content 4.4 and 16.3 times that of FV and AB, respectively (Table 1. The contents of Pb and Cd in the PM are also significantly higher than those in the two FCR (*p <* 0.05) (Table 1).

**Figure 10:**
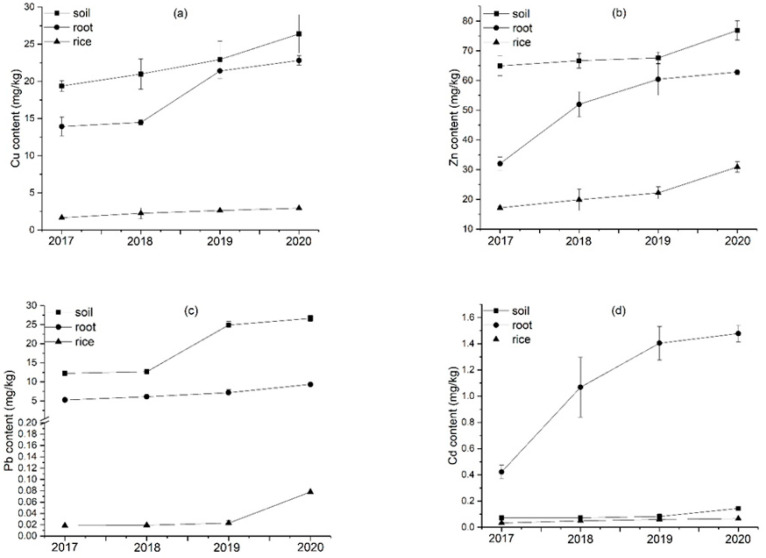
(**a**) Annual increasing rates of Cu content in the soils, rice roots, and brown rice by the application of PM during the four experimental years; (**b**) annual increasing rates of Zn content in the soils, rice roots, and brown rice by the application of PM during the four experimental years; (**c**) annual increasing rates of Pb content in the soils, rice roots and brown rice by the application of PM during the four experimental years; (**d**) annual increasing rates of Cd content in the soils, rice roots, and brown rice by the application of PM during the four experimental years. Data are expressed as means ± SD; *n* = 3.

The inputs of heavy metals into the soils through the application of PM and FCR are much higher than the outputs by the uptake of rice plants, resulting in the annual increase in Cu, Zn, Pb, and Cd contents in the soils for the consecutive four years (Figure 10). The contents of Cu and Zn in the PM soil are significantly higher than CK (*p* < 0.05) during the four years, and the contents of Pb and Cd in the PM soil are also significantly higher than CK (*p* < 0.05) in the latter two years. The contents of Zn and Pb in the AB and FV soils are significantly higher than CK (*p* < 0.05) in the latter two years, and Cd content in the AB and FV soils is also significantly higher than CK (*p* < 0.05) on the fourth years, which is, however, still much lower than in the PM soil (Figure 6). This suggests that the contents of heavy metals in the soils are significantly raised by the application of PM and FCR, and those by the application of PM are more significant. Cu content in the soils of PM annually increases by 8.3%, 9.3%, and 15.0%; Zn content by 2.6%, 1.4%, and 13.7%; Pb content by 3.5%, 96.5%, and 7.0%; Cd content by 0.4%, 14.4%, and 73.3%, respectively.

Pb and Cd are even more rapidly accumulated in the soils than Cu and Zn. The contents of Pb and Cd in the PM soil in the fourth year are 2.2 and 2.0 times that on the first year, while Cu and Zn in the fourth year are 1.4 and 1.2 times that on the first year (Figure 10). It may be because both Cu and Zn are nutrient elements and highly taken up by crops, while Cd and Pb are toxic to and less assimilated by crops and thus are mostly adsorbed by soil particles. Cd is easily accumulated in soil by fertilizer application [45]. Cd in the cultivated layers of soil is significantly raised after the application of PM for 10 years [42,46]. The high content of P in PM may also contribute to the accumulation of toxic metals in the soil as the combination of P with Cd and Pb [47,48].

The content of OM in the soils is significantly correlated with that of Cu, Zn, Pb, and Cd in the soils (r = 0.856, 0.803, 0.928, 0.973, *n* = 12; *p* < 0.01), further suggesting that the accumulation of heavy metals in the soils is mainly attributed to the application of organic manure. On the other hand, the high content of OM is beneficial to the accumulation of heavy metals due to the function of chelation [49,50,51,52]. The higher the content of OM in the soil is, the more Cd is accumulated in the soil [53].

The contents of Cu and Zn in the PM are over the safe limits of organic fertilizers; Pb and C dare below the safe limits (Cu < 100; Zn < 500; Cd < 3; Pb < 100) specified by the China national standards (NY-525-2012) [54]. The risks of heavy metal accumulation caused by the long-term application of PM and FCR can never be neglected. Long-term application of livestock manure, especially PM, is one of the important reasons for the accumulation of heavy metals in farmland [10,25,55,56]. The application of PM results in the enhancement of Cu, Zn, Pb, and Cd contents in soils [57,58,59]. In this study, the annual increments of Cu, Zn, Pb, and Cd in the soil are 2.33, 3.97, 4.80, and 0.024 mg/kg on annual average, respectively. According to the rates of increase, the contents of Cu, Zn, Pb, and Cd in the soil will exceed the safe limits (Cu ≤ 100 mg/kg; Zn ≤ 250 mg/kg; Pb ≤ 140 mg/kg; Cd ≤ 0.60 mg/kg) according to the China national standards (GB15618-2018) [60] after the application of PM for 35, 47, 27, and 22 years.

### 3.3. Distribution of Heavy Metals in Rice Plants

Roots are the passageway of heavy metals from the soil into crops. The contents of Cu, Zn, Pb, and Cd in the rice roots are significantly positively correlated with those in the soils in each experimental year (r = 0.723, 0.707, 0.983, 0.944, *n* = 12; *p* < 0.05), also suggesting that the enhancement of heavy metals in the rice roots is caused by their accumulation in the soils. The contents of Cu, Zn, Pb, and Cd in the rice roots of the three treatments show an annual cumulative trend (Figure 9). The contents of Cu and Zn in the rice roots of PM are significantly higher than CK (*p <* 0.05) during the four years, the content of Pb in the PM rice roots is significantly higher than CK (*p <* 0.05) in the latter two years, and Cd in the PM rice roots is significantly higher than CK (*p <* 0.05) in the latter three years. Cu content in the AB rice roots is significantly higher than CK (*p <* 0.05) in the fourth year, and that in the FV rice roots is significantly higher than CK (*p <* 0.05) in the latter two years. The content of Zn in the rice roots of AB and FV is significantly higher than CK (*p <* 0.05) in the latter three years, Pb content in the rice roots of AB and FV is significantly higher than CK (*p <* 0.05) in the latter two years, and Cd in the AB and FV roots is also significantly higher than CK (*p <* 0.05) in the latter two years (see Figure 7). The contents of heavy metals in the roots of AB and FV, however, are still much lower than those of PM, also suggesting that the application of PM and FCR have made heavy metals accumulate in the rice roots, and the accumulation by the PM is even more significant. Cu content in the rice roots of PM annually increases by 3.8%, 47.9% and 6.7% from 2018 to 2020; Zn by 62.3%, 16.2%, and 4.0%; Pb by 16.3%, 17.4% and 30.4%; Cd by 153.1%, 31.4% and 5.2%, respectively (Figure 7). Especially, Cd content in the rice roots of PM on the fourth year attains as high as 1.48 mg/kg, which is 3.5 times that on the first year and also one order magnitude that in the soils. This suggests that Cd can be actively taken up by the rice roots from the soils.

Heavy metals taken up by the rice roots are partially translocated to the aerial parts. The contents of Cu, Zn, Pb, and Cd in the aerial parts of the rice plants of PM in the fourth year are 22.0%, 50.5%, 8.9%, and 42.2% of the total, respectively. Both Cu and Zn are essential elements for crops and are inclined to be translocated to the rice shoots, thus showing higher rates in the aerial part. Cd and Pb are not only non-essential but also toxic to crops and thus are mostly trapped in the crop roots.

Cu and Zn contents in the different tissues of rice plants are in the decreasing order of roots > grains > stems > leaves and Pb and Cd in the decreasing order of roots > stems > leaves > grains, which are consistent with many previous studies [23,61]. Cu and Zn are mostly accumulated in the roots, reduced in the stems and leaves, and again raised in the rice grains. This indicates that both Cu and Zn, as nutrient elements, can be actively taken up by rice seeds, while Pb and Cd are mainly retained in the rice roots and stems (Figure 9). Only a small amount of Pb is translocated into rice grains, and a relatively higher amount of Cd is translocated into rice grains (Figure 9), which coincides with many previous studies [62,63]. Cd content in the roots and brown rice of PM on the fourth year attains 1.48 mg/kg and 0.066 mg/kg, and Pb content 9.33 mg/kg and 0.078 mg/kg, respectively. The ratio of Cd content in the roots, stems, leaves, and brown rice is 24:16:2:1, compared with that of Pb being 152: 12: 2: 1. This also suggests that Pb is mostly retained in the roots, and Cd is relatively easily translocated into the rice grain.

### 3.4. Risk of Heavy Metal Contamination in Rice Grain Caused by Application of PM and FCR

The contents of Cu, Zn, Pb and Cd in the brown rice of the fertilizer treatments in each experimental year are positively correlated with those in the soils (r = 0.836, 0.745, 0.874, 0.949; *n* = 12; *p* < 0.01) and rice roots (r = 0.843, 0.930, 0.926, 0.968, *n* = 12; *p* < 0.01). The contents of Cu and Zn in the brown rice of PM, FV, and AB in the latter two years are significantly higher than CK (*p* < 0.05) (Figure 8). Moreover, Cu content in the brown rice of PM, FV, and AB in the latter two years is significantly higher than that in the first two years (*p* < 0.05). Pb content in the brown rice of PM, AB, and FV in the fourth year is significantly higher than CK (*p* < 0.05), and Cd content in the brown rice of PM and FV in the four years is significantly higher than CK (*p* < 0.05). Likewise, the contents of Cu, Zn, Pb, and Cd in the brown rice of AB and FV are much lower than those of PM. Heavy metals in the brown rice of the PM treatment are annually raised more significantly than those of AB and FV (*p* < 0.05) (Figure 8). For example, Cu content in the brown rice of PM attains 2.94 mg/kg in the fourth year, which is 2.0, 1.1, and 1.2 times that of CK, AB, and FV, respectively. This also suggests that the application of PM and FCR has already posed a risk of heavy metal accumulation in brown rice, and the risk caused by the application of PM is much higher.

A root/rice ratio of Cd content in the PM rice plants is 22.1, and that of Pb is 119.7, suggesting that Cd is more easily translocated into the rice grains and Pb is mostly retained in the rice roots, which is consistent with many previous studies [64,65,66]. It was also reported that 10% of rice samples randomly collected in the market in China exceed the safe limit of Cd content (Cd ≤ 0.2 mg/kg) specified by the China national standards [67]. Cd accumulation in soil has posed a threat to human health through the food chain [68,69].

The contents of heavy metals in the brown rice of the treatments for the consecutive four years are still below the safe limits, specified by the China national standards (GB 2762-2017, NY861-2004) [70,71]. Zn in rice grains inhibits the further uptake of Cd by grains to protect itself from toxicity [72,73]. The high contents of Cu and Zn in rice grains reduce the mobility of Pb and Cd in rice plants [74].

However, Cu content in the brown rice of PM increases by 35.5%, 16.7%, and 12.7% annually from 2018 to 2020; Zn increases by 15.7%, 11.5%, and 39.2%; Pb increases by 4.4%, 18.8% and 141.9%; Cd increases by 48.0%, 21.3%, and 12.0%, respectively (Figure 10). The amount of annual increase in Cu, Zn, Pb, and Cd in the brown rice of PM is 0.43, 4.56, 0.019, and 0.011 mg/kg on average, respectively. If continuously increasing at the rates, the contents of Cu, Zn, Pb, and Cd in the rice grains will exceed the safe limits (NY861-2004, Cu ≤ 10 mg/kg; Zn ≤ 50 mg/kg; Pb ≤ 0.40 mg/kg; Cd ≤ 0.20 mg/kg) [71] after 20, 8, 21 and 16 years of the application of pig manure, respectively. Long-term application of PM to paddy fields raises the risk of Cd contamination of rice [75]. The risk of heavy metal accumulation in rice grains by long-term application of PM and FCR, therefore, can never be neglected.

**Figure 11:**
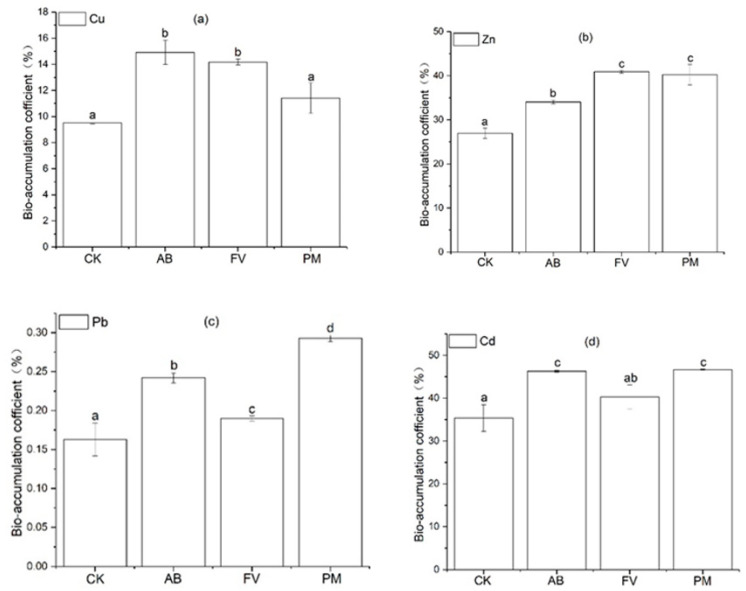
(**a**) BAC of Cu in the brown rice of the different treatments; (**b**) BAC of Zn in the brown rice of the different treatments; (**c**) BAC of Pb in the brown rice of the different treatments; (**d**) BAC of Cd in the brown rice of the different treatments. Data are expressed as means ± SD; *n* = 3. Different letters on data in the same group indicate a significant difference between them at *p* < 0.05 level.

The bioaccumulation coefficients (BAC) in rice, brown rice/soils ratios of heavy metals were calculated. Cd is much mobile in grain plants and can be actively taken up by grain [76,77,78]. The BAC of Cd in the rice grains is the highest (Figure 11). Cu and Zn are two essential elements for plants and can also be easily taken up and transferred to rice grain. Thus, the BAC of Cu and Zn in the grains is also high. Pb is mostly retained in the roots, and its BAC is the lowest. The BAC of heavy metals for the AB, FV, and PM treatments are mostly significantly higher than CK (*p* < 0.05), suggesting that the applications of PM and FCR increase the risk of heavy metal accumulation in rice grains. Moreover, the BAC of Pb in the PM grains is significantly higher than that in CK, AB, and FV (*p* < 0.05), the BAC of Zn in the PM and FV grains significantly higher than that in CK and AB, and the BAC of Cd in the PM and AB grains significantly higher than that in CK and FV (*p* < 0.05), further suggesting that the application of PM more easily leads to the accumulation of heavy metals in rice grains.

## 4. Materials and Methods

### 4.1. Study Area

Located in front of the Yangtze River Delta, Shanghai is surrounded by water on three sides. The land area of Shanghai was initially created by the combined action of the Yangtze River and the East China Sea, with an elevation of 3.2 m on average. Shanghai has a subtropical humid monsoon climate with a mean annual temperature of 17.1 °C and a mean annual rainfall of 1223.4 mm. An experimental field is situated at Professor’s Farm (31 07′ N, 120 53′ E) of Qingpu District in the western suburb of Shanghai. The soils in the experimental site are mostly derived from the sediments of lakes and rivers, with clayey loam texture, and are mostly classified as entisols. Some physical-chemical properties of the experimental soil are indicated in Table 2.

### 4.2. Field Experiment

Field experiments were carried out for four consecutive years from 2017 to 2020. In each experimental year, the same four fertilizer treatments were arranged, which included the applications of PM, residues of AB and FV as well as non-fertilizer control (CK). Each treatment is triplicated, and the number of experimental plots is 12 in total, with each plot being 2.5 m × 10 m in area and an interval of 50 cm between the two plots.

Rice seedlings were cultivated on seedbed in late May and then transplanted to the experimental fields in middle June. Rice grains were harvested in late October. The whole period of rice growth in the experimental years is about 120 days. A local variety of rice, Jiahe 218, suitable to the climate in Shanghai, was selected for the experiment.

PM, taken from a local pig farm, was composted for several months before being applied to the plots as an experimental treatment. Likewise, the composed culturing residues of AB and FV fetched from the two companies of edible fungi production were also applied as experimental treatments. The culture medium of FV is originally composed of corncob, hull of cottonseeds, rice bran, and husk of wheat, and that of AB composed of straw, chicken manure, rapeseed cake, hull of cottonseeds, and soybean pulp. The contents of nutrients and heavy metals in the two FCR are indicated in Table 1.

Each experimental treatment included basal and topdressing fertilizations, which were applied before seedling transplanting in early June, and during seedling jointing in early July, respectively. The amount of fertilizer application for each treatment is calculated based on the equivalent N content applied by conventional rice farming during the whole rice-growing season, as shown in Table 3.

### 4.3. Investigation and Sampling

The growth of rice plants was investigated each week during the whole rice-growing season. The rice grains of each plot were harvested and weighed independently to calculate the actual yield of rice grains.

Six rice plants in each plot were randomly collected in each experimental plot after it was matured. The plant samples were immediately carried to the laboratory, cleaned with deionized water, and divided into the subsamples of roots, stems, leaves, and rice grains, which were oven-dried at 90 °C for 30 min and then continuously dried at 70 °C for 12 h. The dried plant samples were ground into powder for chemical analyses.

The cultivated layers of the soils (0–20 cm) in each plot were sampled in the different periods of the rice-growing season. The samples were crushed, ground, and then passed through 2 mm- and 0.149 mm-sized nylon sieves successively for chemical analyses.

### 4.4. Sample Analyses

The content of OM in the soils was determined by the potassium dichromate-sulfuric acid method; TN by the Kjeldahl method; AN by the alkaline diffusion method; TP by the acid digestion-phosphomolybdate colorimetric method; AP by the NaHCO_3_ solution extraction-phosphomolybdate colorimetric method; TK content by the acid dissolution method; AK by ammonium acetate extraction method [79].

0.2 g soil sample (<0.149 mm) was put into Teflon crucible and digested with the three mixed acids (HNO_3_-HClO_4_-HF). All the reagents are guaranteed. The contents of Cu and Zn in the solution were determined by the inductively coupled plasma-atomic emission spectrometry (ICP-AES, Mason, OH, USA) of Leeman Prodigy produced by American Leeman Instrument Co. Ltd., with the error being 1.52–3.43%, and the relative standard deviation being less than 3%. The minimum detection limits for Cu and Zn are 0.002 µg/mL. The contents of Cd and Pb were determined by the graphite furnace atomic absorption spectrophotometer (GF-AAS, Jena, Germany) (Zee nit 600/650) produced by Jena Company, Germany, with the error being 2.31–4.13% and the relative standard deviation being less than 5%. The minimum detection limits for Pb and Cd are 0.06 µg/L and 0.008 µg/L.

The samples of rice plants, PM, and FCR were put into beakers and digested with the mixed acids (HNO_3_ + HCl + HClO_4_). The contents of Cu, Zn, Cd, and Pb in the solution were determined by the GF-AAS.

The bioavailability of heavy metals in soils was evaluated by BAC. The BAC (%) of a heavy metal element in rice grains reflects the transfer and bioaccumulation of the element from soil to grain [80].

(1) $$\mathrm{BAC}(\%)=\frac{C_{\mathrm{rice}}}{C_{x}}\times 100$$

C_rice_: Heavy metal content in rice grains; C_x_: heavy metal content in soil.

### 4.5. Statistical Analysis

All the experimental data were firstly treated by Microsoft Excel 2016 (Microsoft, Redmond, WA, USA). The data were commonly expressed as the mean ± standard deviation (SD), and the statistical difference was checked by the Duncan test, with the significance level set to *p* < 0.05. The significances of differences among the data and Pearson correlations between the two groups of data were analyzed by IBM SPSS Statistics 24.0 (IBM, New York, NY, USA). All the graphs were drawn using Origin 9.1 (OriginLab, Northampton, MA, USA).

## 5. Conclusions

The applications of PM and FCR to the paddy fields raise the contents of OM and nutrients in the soils significantly and increase grain yield effectively. However, both lead to an annual accumulative increase in heavy metals in the soils. Especially, the risk of heavy metal accumulation by the application of PM is more significant. Cu and Zn contents in the soil of the PM treatment are significantly raised during the four years, and Pb and Cd are also significantly raised in the latter two years.

Heavy metals in the rice plants by the application of PM and FCR also increase annually. These taken up by plants are mostly retained in the roots. Cu and Zn contents in the rice roots of PM are significantly higher than CK during the four experimental years, and Pb and Cd are also significantly higher than CK in the latter two years. Cu and Zn contents in the rice plants are in the decreasing order of roots > rice grains > stems > leaves, and Pb and Cd in the decreasing order of roots > stems > leaves > rice grains.

The contents of Cu, Zn, Pb, and Cd in the brown rice by the PM, FV, and AB treatments increase annually, which are positively significantly correlated with those in the soils and rice roots (*p* < 0.05). This suggests that the heavy metals accumulated in the brown rice are caused by the applications of PM and FCR. The risk of heavy metals by the application of PM is even more significant. Though the contents of heavy metals in the brown rice are still within the safe levels during the experimental years, the risks of their accumulative increments, especially by long-term application of PM, can never be neglected.

## Figures and Tables

**Table 1:** Nutrient and heavy metal content of PM and FCR in field experiments. Data are means ± SD, *n* = 3. Different letters indicate significant differences between treatments at *p* < 0.05 according to the Duncan test.

Organic Fertilizer	pH	OM (%)	Water Content (%)	TN (g/kg)	TP (g/kg)	TK (g/kg)	Cu (mg/kg)	Zn (mg/kg)	Pb (mg/kg)	Cd (mg/kg)
FV	5.50 ± 0.11	71.75 ± 0.89	77.66 ± 0.13	20.34 ± 0.26	10.20 ± 0.09	3.07 ± 0.07	8.00 ± 0.81c	33.46 ± 5.95c	0.52 ± 0.16 c	0.15 ± 0.05 c
AB	5.85 ± 0.16	36.49 ± 0.63	75.63 ± 0.12	19.24 ± 0.31	4.36 ± 0.05	2.65 ± 0.15	30.09 ± 0.49b	123.72 ± 6.52b	1.71 ± 0.43 b	0.30 ± 0.05 b
PM	6.99 ± 0.19	33.81 ± 0.55	12.04 ± 0.15	21.05 ± 0.18	15.43 ± 0.16	3.76 ± 0.21	166.76 ± 19.99a	544.74 ± 75.48a	3.20 ± 0.14 a	0.43 ± 0.03 a

**Table 2:** Background values of nutrients in the experimental soil in the suburb of Shanghai, Southeast China. Data are means ± SD, *n* = 3.

pH	OM (g/kg)	TN (g/kg)	TP (g/kg)	TK (g/kg)	AN (g/kg)	AP (g/kg)	AK (g/kg)
5.20 ± 0.12	31.07 ± 0.28	1.41 ± 0.21	0.14 ± 0.02	17.91 ± 0.48	0.11 ± 0.005	0.006 ± 0.0004	0.044 ± 0.006

**Table 3:** Quantities of base fertilizer and topdressing to the rice of the field experiment.

	Base Fertilizer (June 2017–June 2020)					Topdressing (July 2017–July 2020)			
Treatment	Fertilization Amount	TN (g/m^−2^)	TP	TK		Fertilization Amount	TN	TP	TK
	(kg/m^2^)	(g/m^2^)	(g/m^2^)	(g/m^2^)		(kg/m^2^)	(g/m^2^)	(g/m^2^)	(g/m^2^)
CK	0.00	0.00	0.00	0.00	CK	0.00	0.00	0.00	0.00
AB	1.33	9.50	2.15	0.65	AB	3.80	27.6	10.88	5.84
FV	0.57	9.50	3.36	1.14	FV	4.04	27.6	13.75	4.91
PM	0.48	9.50	5.52	1.15	PM	1.21	27.6	16.99	3.03

## Data Availability

All data are provided in the manuscript.
